# L-shaped association between dietary iron intake and HPV infection: a cross-sectional analysis based on national health and nutrition examination survey 2005–2016

**DOI:** 10.3389/fnut.2025.1530624

**Published:** 2025-02-11

**Authors:** Xiaotong Chen, Haiwei Chen, Yuling Chen, Lixin Tang, Jiaqi Liu, Yu-Hua Ou

**Affiliations:** ^1^Department of Clinical Medicine, The Second Clinical College of Guangzhou Medical University, Guangzhou, China; ^2^Department of Gynecology, The Second Affiliated Hospital of Guangzhou Medical University, Guangzhou Medical University, Guangzhou, China

**Keywords:** human papillomavirus, iron intake, nutrition, female reproductive diseases, infectious diseases

## Abstract

**Background:**

Human Papillomavirus (HPV) infection is a ubiquitous sexually transmitted infection globally, frequently associated with sexual behaviors characterized by increased frequency and multiple partnerships. The relationship between varying levels of dietary iron intake and the occurrence of Human Papillomavirus (HPV) infection remains an unresolved question in the scientific community. The objective of our study was to investigate the potential relationship between the consumption of dietary iron and HPV infection.

**Methods:**

Our investigation drew upon comprehensive datasets from 7,819 participants enrolled in the National Health and Nutrition Examination Survey (NHANES) from 2005 to 2016. Employing a cross-sectional analytical framework, we delved into the potential correlation between dietary iron consumption and Human Papillomavirus infection. To statistically assess this relationship, we utilized weighted multivariate logistic regression models. Additionally, we implemented smooth curve fitting and threshold effect analysis, to delineate the complex, nonlinear association between iron intake and HPV infection. Furthermore, we conducted subgroup analyses.

**Results:**

After adjusting for multiple confounding variables, our results demonstrated a statistically significant inverse association between iron intake and HPV infection (OR = 0.988, 95% CI: 0.979–0.998, *p* = 0.018). It’s worth noting that, in comparison to individuals in the quartile with the lowest iron intake, those in the highest quartile exhibited a 23.2% reduction in the odds of HPV infection for each incremental unit of iron intake (OR = 0.768, 95% CI: 0.634 to 0.930, *p* = 0.009). A refined analysis employing smoothing curve fitting techniques unveiled an L-shaped correlation, delineating a specific relationship between dietary iron intake and the incidence rate of Human Papillomavirus infection. When iron intake was <16.99 mg, a higher incidence of HPV infection was associated with lower levels of iron intake. (OR = 0.968, 95% CI: 0.956–0.980, *p* < 0.001).

**Conclusion:**

The presence of an L-shaped association between iron intake and HPV infection underscores and emphasizes the possible beneficial effect of sufficient iron intake in reducing the likelihood of HPV infection.

## 1 Introduction

Human Papillomavirus (HPV) infection constitutes a prevalent sexually transmitted infection on a global scale, posing a significant public health challenge. Structurally, HPV is characterized by its small, circular double-stranded DNA genome, classified into high-risk and low-risk genotypes. The low-risk genotypes, notably HPV-6 and HPV-11, are commonly associated with benign conditions such as genital warts (1). Chronic infection with high-risk HPV genotypes has been conclusively linked to a significant rise in cervical cancer cases among women (2). Currently, twelve specific HPV subtypes—HPV 16, 18, 31, 33, 35, 39, 45, 51, 52, 56, 58, and 59—are recognized as high-risk and are implicated in the oncogenesis of cervical cancer in women (3). Of particular significance, HPV16 and HPV18 have been identified as the primary subtypes that are closely associated with the onset of cervical cancer in a substantial proportion, exceeding 70%, of women worldwide (4, 5). Despite notable advancements achieved through HPV vaccination in mitigating the risk of infection and consequently reducing cervical cancer incidence, the vaccine’s efficacy remains confined in terms of its ability to cover high-risk HPV strains and specific variant subtypes (6, 7). Consequently, in the contemporary post-vaccination context, a more expansive and multifaceted approach to HPV infection prevention may necessitate interventions that prioritize the alteration of individuals’ lifestyles and dietary practices.

An indispensable trace element, iron occupies a pivotal role in a multitude of bodily functions. Within human physiology, it is particularly crucial for the biosynthesis of deoxyribonucleic acid (DNA) and the facilitation of oxygen transportation mechanisms (8, 9). Approximately 65% of the total body iron is sequestered within the hemoglobin molecules of red blood cells. Conversely, a minor proportion, constituting about 10% of the total iron pool, is allocated to myoglobin in muscular tissues and is also incorporated into a variety of enzymes and cytochromes present in diverse tissue compartments (10). Typically, adults experience a daily excretion of iron in the range of 1 to 2 milligrams (1–2 mg). In adult females, an augmented amount of iron is lost during the menstrual cycle. Non-menstrual iron loss primarily stems from the desquamation of the gastrointestinal mucosa and epidermal cells, alongside minor hemorrhagic occurrences (9). Given the absence of an endogenous excretory mechanism for iron, the human organism relies on dietary management to sustain iron homeostasis (11). The preservation of iron concentrations within a tightly controlled physiological range is paramount. Deviations from this optimal range, whether from iron deficiency or iron overload, can significantly impair physiological integrity, potentially precipitating a spectrum of pathological conditions and adverse health sequelae (10, 12).

Recent research has highlighted the paramount importance of maintaining an optimal iron balance, which is indispensable for cancer initiation, progression, therapeutic responsiveness, and immune system competence (13, 14). A substantial body of research has indicated that increased iron levels may promote the formation of free radicals, augmenting oxidative stress responses and potentially accelerating the development of oncogenic transformations (15–17). Dietary iron-laden foods, such as red and processed meats, have been correlated with a heightened cancer risk in several epidemiological studies (18–21). Specifically, dietary iron overload leading to hyperferremia has been identified as a significant predictor of breast cancer, especially in postmenopausal women (22). Conversely, iron deficiency within the body has also been implicated in accelerating cancer progression (23, 24). This may be attributed to iron deficiency impeding ferroptosis, a form of iron-dependent regulated cell death induced by lipid peroxide accumulation, which subsequently disrupts immune surveillance mechanisms (25–28). Currently, there is an active pursuit of anticancer therapies targeting ferroptosis-related pathways in cancer cells (14). In light of iron’s dual impact, establishing an optimal dietary iron intake is crucial for systemic iron regulation.

While both iron excess and deficiency have been implicated in various cancers, the relationship between iron intake and cervical cancer caused by human papillomavirus (HPV) infection remains an area of ongoing investigation. Research undertaken by Elham Nazari unveiled a beneficial impact of consuming iron through diet on mitigating the incidence and advancement of cervical cancer among Iranian females (29). Conversely, a recent observational investigation suggested that females exhibiting elevated ferritin levels demonstrated a reduced likelihood of experiencing resolution of oncogenic human papillomavirus (HPV) infections, in comparison to those with lower ferritin concentrations (30).

Notwithstanding these discoveries, the present body of evidence linking iron intake to HPV infection remains unresolved and lacks definitive conclusions (31). Therefore, we conducted an analysis of data from the National Health and Nutrition Examination Survey (NHANES) to evaluate the potential correlation between iron intake and HPV infection. Our objective was to identify a balanced iron intake level that could provide dietary guidance for preventing the onset of HPV infection in women.

## 2 Materials and methods

### 2.1 Data provenance and examined population

**Figure 1:**
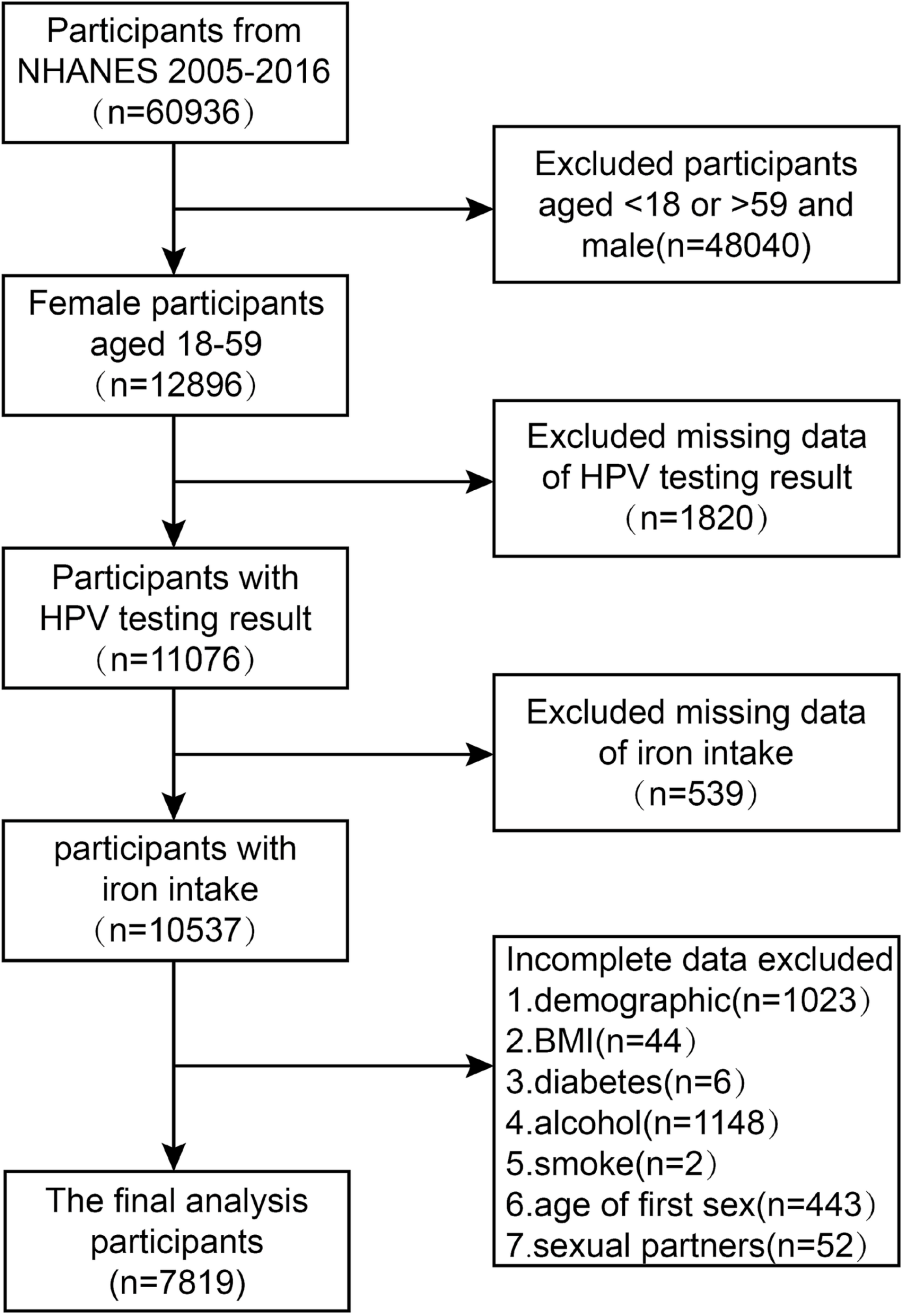
Flowchart of the sample selection from NHANES 2005–2016.

Our research endeavor employed cross-sectional data obtained from six sequential iterations of the National Health and Nutrition Examination Survey (NHANES), encompassing the timeframes from 2005–2006 to 2015–2016, with a comprehensive sample of 60,936 participants. NHANES represents a substantial, population-representative cross-sectional study executed by the National Center for Health Statistics (NCHS), a division of the United States Centers for Disease Control and Prevention (CDC). Rigorous adherence to the NHANES’ prescribed guidelines and protocols was maintained throughout all analytical procedures conducted within the scope of our investigation. Upon the conclusion of an exhaustive and rigorous exploration and screening protocol applied to the NHANES database, a cohort of 60,936 subjects spanning the years 2005 to 2016 was initially deemed eligible for inclusion in this investigative study. After this initial identification phase, the sample was refined to encompass exclusively 12,896 female participants aged 18 to 59 years. Exclusion criteria were applied, resulting in the removal of participants with missing data on HPV infection status (1820 subjects), iron intake (539 subjects), or incomplete covariate information (2,718 subjects). Ultimately, the resultant analytical sample consisted of 7,819 subjects. A schematic diagram, presented in Figure 1, illustrates the systematic process of participant screening and inclusion in this study.

### 2.2 Variables

The research endeavor was centered on two key exposure variables: iron intake and human papillomavirus (HPV) infection. Data on dietary intake were obtained through a two-day, non-consecutive dietary recall protocol employed by NHANES. Specifically, the first dietary recall interview took place face-to-face at a mobile examination center (MEC), whereas the subsequent interview was administered telephonically within a window of 3 to 10 days thereafter. Owing to the shortcomings observed in the second phase of interviews, our analysis relied on iron intake figures sourced from the first phase of food consumption data. To estimate dietary iron nutrition, NHANES referred to the authoritative Dietary Research Food and Nutrition Database maintained by the United States Department of Agriculture. It should be clarified that the dietary iron intake information gathered in this study exclusively reflected the amounts ingested from food sources, excluding any contribution from iron supplements. For the conduct of statistical analysis, the iron intake values were stratified into quartiles: Q1 spanning 0 to 8.16 milligrams per deciliter (mg/dL), Q2 ranging from 8.17 to 11.73 mg/dL, Q3 encompassing 11.74 to 16.39 mg/dL, and Q4 extending from 16.4 to 82.99 mg/dL. In the context of the NHANES repository, the ascertainment of HPV infection status necessitated the collection of vaginal cellular material using a swab, followed by the isolation and analysis of DNA from the swab sample with the aid of the Roche Prototype Line Blot Test and the Roche Linear Array (LA) Human Papillomavirus Genotyping Assay. Participants whose test results were “positive” were classified as HPV-positive, whereas those yielding “negative” results were deemed HPV-negative.

### 2.3 Other covariates

Utilizing existing literature and clinical insights, we incorporated a range of covariates that might modulate the correlation between iron intake and HPV infection. The continuous variables integrated into this investigation encompassed age, poverty-to-income ratio (PIR, Household or individual income divided by poverty guidelines for a given survey year), and body mass index (BMI, Divide the weight of an individual by the square of its height, kg/m^2^.). The categorical variables included race/ethnicity, education level, marital status, alcohol consumption (at least 12 drinks per year?), diabetes history (have you been told by a doctor or health professional that you have diabetes?), history of smoking (at least 100 cigarettes in a lifetime?), age of first sex, and number of sexual partners in the past year. Comprehensive details regarding the aforementioned covariates can be accessed on the NHANES website.

### 2.4 Analytical statistics

We performed a descriptive statistical analysis of the variables in our study by computing metrics such as the mean, median, and standard deviation for each variable listed in the baseline table. This approach facilitated the identification of trends in data concentration and dispersion. To elucidate the demographic characteristics of patients with and without HPV infection, we utilized ANOVA to assess the statistical significance of differences in continuous variable means and the chi-square test to compare differences in categorical variable frequency distributions. Continuous variables were reported using mean ± standard deviation (SD), while categorical variables were presented as frequency (percentage). Notably, given the complex sampling design of the NHANES database, we employed weighted univariate analysis to correct for sampling bias and differences in sampling probabilities across populations, thereby minimizing sampling error and enhancing the precision and external validity of our estimates. Adopting statistical techniques involving weighted univariate and multivariate logistic regression frameworks, we embarked on an investigation to scrutinize the connection between iron ingestion and the occurrence of HPV infection. In the multivariate logistic regression analyses, we constructed three models to evaluate the statistical relationships of various factors with HPV infection. Specifically, we developed a binary logistic model with HPV infection status (infected/uninfected) as the dependent variable and iron intake and other demographic characteristics as independent variables. We calculated the adjusted odds ratios (AORs) for the following models: (1) Model 1 (unadjusted for covariates), (2), Model 2 (adjusted for age and race), and (3) Model 3 (adjusted for all covariates) to ascertain the link between iron intake and HPV infection. Furthermore, based on the fully adjusted models, we conducted subgroup analyses using multilevel multivariate logistic regression to identify stratified associations between iron intake and HPV infection. Forest plots were generated to facilitate interaction tests among subgroups. To analyze the relationship between iron intake and HPV infection, we considered threshold and saturation effects and incorporated a threshold variable to categorize iron intake into two groups: below and above the threshold. Logistic regression models were employed to assess the impact of iron intake on HPV infection. By comparing the odds ratios (ORs) and 95% confidence intervals (CIs) between these two groups, we observed changes in the risk of HPV infection. Additionally, smooth curve fitting was applied in the fully adjusted model to visually estimate the association between iron intake and HPV infection. In our study, statistical significance was determined at a *p*-value of <0.050.

## 3 Results

### 3.1 Baseline features

A sample of 7,819 adults aged 18 to 59 years was enrolled in this study, with 3,481 individuals (constituting 44.52% of the cohort) diagnosed with HPV infection. Table 1 presents a detailed demographic and behavioral profile of the participants, stratified by their HPV infection status. Notably, non-Hispanic Black women who were divorced, older, economically disadvantaged, less educated, sexually active at an earlier age, and with histories of smoking, alcohol abuse, and diabetes mellitus exhibited an elevated prevalence of HPV infection. The mean dietary iron intake among participants with a confirmed diagnosis of HPV infection was 12.68 ± 0.18 milligrams per day, which was statistically significantly lower (*p* < 0.050) compared to the mean intake of 13.67 ± 0.17 milligrams per day observed in those without HPV infection. When the cohort was stratified into quartiles based on their dietary iron intake levels, a statistically significant difference (p < 0.050) in HPV infection status persisted between the quartiles.

**Table 1:** Baseline characteristics of the study population.

Characteristics	HPV infection		*p*- value
	Yes (*n* = 3,481)	No (*n* = 4,338)	
Age, years, Mean (SD)	38.38 (0.37)	41.37 (0.27)	<0.001
Poverty-to-income ratio, Mean (SD)	2.69 (0.05)	3.24 (0.05)	<0.001
BMI, kg/cm^2^, Mean (SD)	28.98 (0.19)	28.91 (0.18)	0.755
RACE, *n* (%)			<0.001
Mexican American	495 (7.80%)	768 (8.62%)	
Other Hispanic	351 (6.32%)	418 (5.00%)	
Non-Hispanic White	1,378 (62.63%)	1984 (70.97%)	
Non-Hispanic Black	1,033 (18.02%)	670 (7.62%)	
Other Race - Including Multi-Racial	224 (5.23%)	498 (7.78%)	
Education level, *n* (%)			<0.001
Less than high school	1,472 (36.93%)	1,541 (29.62%)	
High school and above	2009 (63.07%)	2,797 (70.38%)	
Marital status, *n* (%)			<0.001
Married	1952 (58.16%)	3,309 (78.66%)	
Divorce	567 (17.58%)	373 (8.76%)	
Separated	187 (4.34%)	138 (2.28%)	
Never married	775 (19.92%)	518 (10.30%)	
The doctor told you have diabetes, *n* (%)			0.014
Yes	231 (4.69%)	316 (6.16%)	
No	3,250 (95.31%)	4,022 (93.84%)	
Smoked at least 100 cigarettes in life, n (%)			<0.001
Yes	1,541 (47.26%)	1,394 (35.90%)	
No	1940 (52.74%)	2,944 (64.10%)	
Had at least 12 alcoholic drinks 1 year, *n* (%)			<0.001
Yes	2,549 (79.31%)	2,780 (71.99%)	
No	932 (20.69%)	1,558 (28,01%)	
How old when first had sex, *n* (%)			<0.001
<18	2,321 (67.73%)	2,185 (51.18%)	
> = 18	1,160 (32.27%)	2,153 (48.82%)	
Male sex partners/year, *n* (%)			<0.001
0–1	2,706 (78.16%)	4,089 (94.61%)	
2–5	714 (20.44%)	236 (5.08%)	
>5	61 (1.40%)	13 (0.30%)	
Iron intake, mg, Mean (SD)	12.68 (0.18)	13.67 (0.17)	0.001
Iron intake (quartile), *n* (%)			<0.001
Q1 (0–8.16)	991 (27.91%)	957 (21.51%)	
Q2 (8.17–11.73)	905 (27.92%)	1,050 (23.84%)	
Q3 (11.74–16.39)	801 (21.59%)	1,159 (27.58%)	
Q4 (16.4–82.99)	784 (22.58%)	1,172 (27.07%)	

Mean (SD) for continuous variables, % for categorical variables. BMI, body mass index.

### 3.2 Correlation between iron intake and HPV infection

The outcomes of the multivariate regression analysis, which investigates the relationship between iron intake and HPV infection, are displayed in Table 2. Across three distinct models—an unadjusted model, a partially adjusted model, and a fully adjusted model—a significant inverse relationship was identified between elevated levels of iron consumption and the likelihood of HPV infection, with statistical significance. In the unadjusted model, OR was 0.981 (95% CI: 0.972–0.991, *p* < 0.001), indicating a decrement in the risk of HPV infection with each incremental increase in iron intake. This inverse relationship persisted in both the partially adjusted model (OR = 0.983, 95% CI: 0.973–0.992, p < 0.001) and the fully adjusted model (OR = 0.988, 95% CI: 0.979–0.998, *p* = 0.018).

**Table 2:** Association between iron intake and HPV infection.

	Model 1		Model 2		Model 3	
	OR (95% CI)	*p* value	OR (95% CI)	*p* value	OR (95% CI)	*p* value
Iron intake (mg)	0.981 (0.972, 0.991)	<0.001	0.983 (0.973, 0.992)	<0.001	0.988 (0.979, 0.998)	0.018
Iron intake quartile						
Q1 (0–8.16)	1.00 0(1.000, 1.000)	1.000	1.000 (1.000, 1.000)	1.000	1.000 (1.000, 1.000)	1.000
Q2 (8.17–11.73)	0.903 (0.772, 1.055)	0.201	0.919 (0.784, 1.078)	0.303	0.974 (0.822, 1.154)	0.760
Q3 (11.74–16.39)	0.603 (0.509, 0.715)	<0.001	0.627 (0.530, 0.743)	<0.001	0.712 (0.597, 0.850)	<0.001
Q4 (16.4–82.99)	0.643 (0.542, 0.762)	<0.001	0.663 (0.559, 0.787)	<0.001	0.768 (0.634, 0.930)	0.009
*p* for trend		<0.001		<0.001		<0.001

Model 1 adjusted for no covariates. Model 2 adjusted for age and race. Model 3 adjusted for age, race, poverty-to-income ratio, education level, marital status, body mass index, diabetes history, alcohol consumption, history of smoking, age of first sex, and number of sexual partners.

When iron intake was stratified into quartiles, Q3 and Q4 demonstrated a statistically significant negative correlation with HPV infection compared to Q1 in all three models (*p* < 0.05). In the fully adjusted model, a comparison of the highest quartile to the lowest quartile revealed a 23.2% decrement in the risk of HPV infection for each incremental unit of iron intake (OR = 0.768, 95% CI: 0.634–0.930, *p* = 0.009).

**Figure 2:**
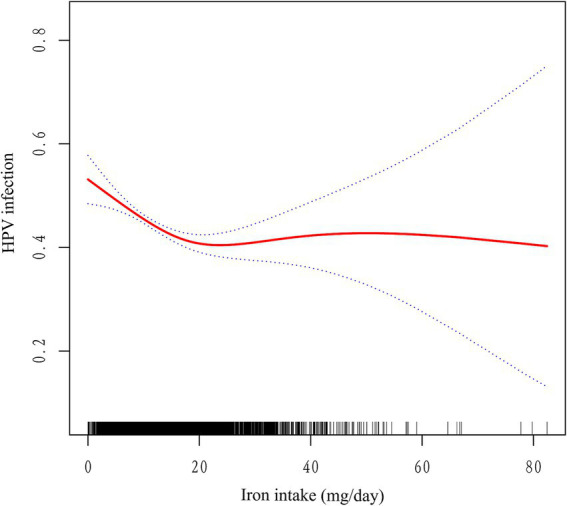
Association between iron intake and HPV infection. The red solid line indicates the smooth curve fit between the variables. The blue dashed line indicates the 95% confidence interval of the fit. Adjusted for age, race, poverty-to-income ratio, education level, marital status, body mass index, diabetes history, alcohol consumption, history of smoking, age of first sex, and number of sexual partners.

To conduct a more thorough examination of the potential association between iron consumption and human papillomavirus (HPV) infection, we conducted an analysis utilizing smooth curve fitting techniques. The statistical model employed in this analysis incorporated comprehensive adjustments for a multitude of covariates, including age, race, poverty-to-income ratio, education level, marital status, body mass index, diabetes history, alcohol consumption, history of smoking, age of first sex, and number of sexual partners. The findings from this analysis revealed a significant L-shaped correlation between increasing levels of iron intake and the risk of HPV infection (Figure 2). The results from an augmented threshold effect analysis, as detailed in Table 3, indicate the presence of a saturation threshold for iron intake at an inflection point of 16.99 milligrams. Below this threshold, a statistically significant inverse relationship is observed, where each incremental unit of iron intake is associated with a 3.2% decrement in the odds of HPV infection (OR = 0.968, 95% CI: 0.956–0.980, *p* < 0.001). Conversely, above this inflection point, no significant correlation is discernible between further increments in iron intake and the prevalence of HPV infection (OR = 1.006, 95% CI: 0.993–1.018, *p* = 0.372), suggesting a ceiling effect on the protective benefits of iron against HPV infection.

**Table 3:** Threshold effect analysis of iron intake and HPV infection.

	Adjusted OR (95% CI)	*p* value
Fitting by the 2-piecewise linear model		
Inflection point	16.99	
Iron intake<16.99	0.968 (0.956, 0.980)	<0.001
Iron intake> = 16.99	1.006 (0.993, 1.018)	0.372
*p* for Log-likelihood ratio	<0.001	

Adjusted for age, race, poverty-to-income ratio, education level, marital status, body mass index, diabetes history, alcohol consumption, history of smoking, age of first sex, and number of sexual partners.

### 3.3 Subgroup analysis

**Figure 3:**
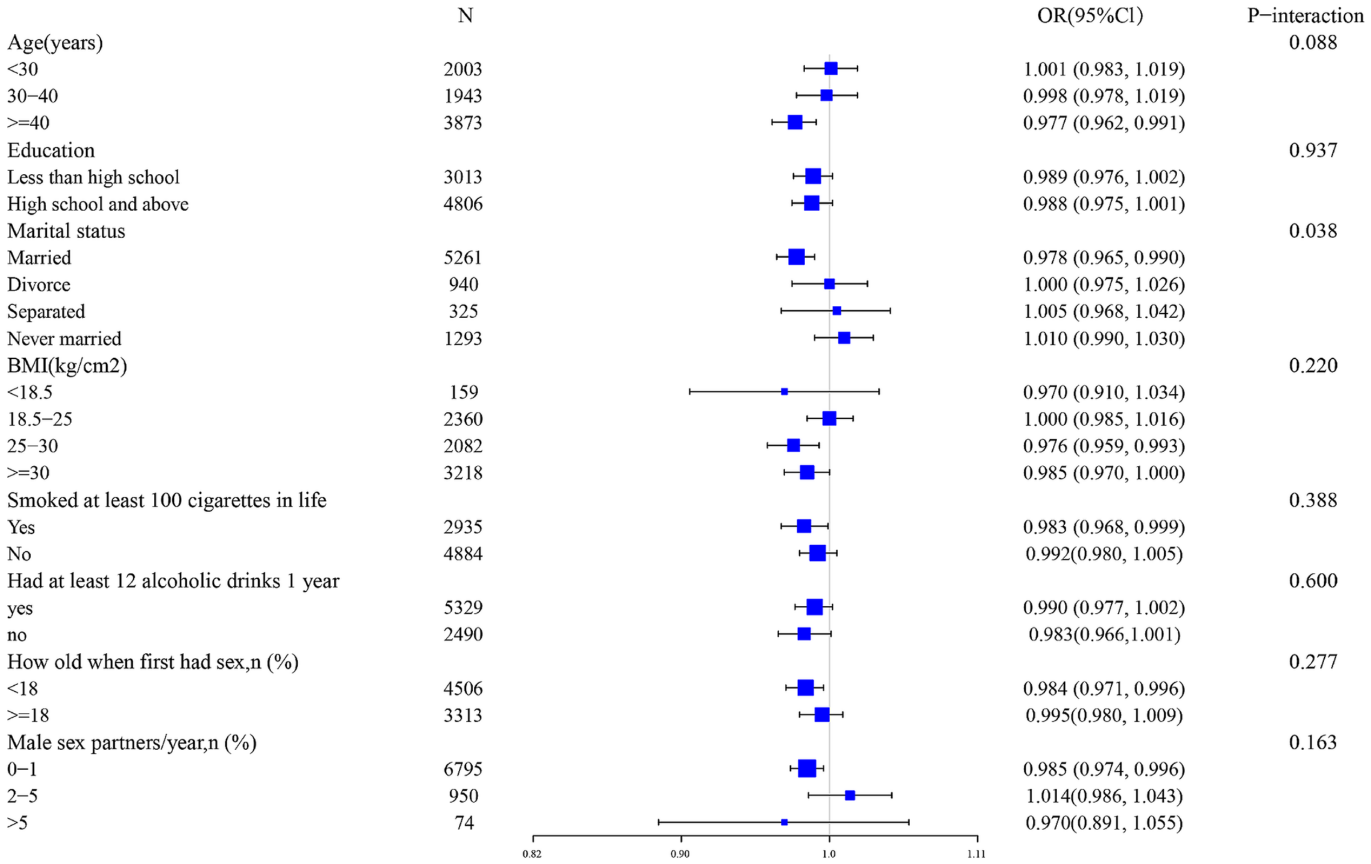
Subgroup analysis of the association between iron intake and HPV infection.

Ultimately, we conducted an extensive series of subgroup analyses. Our cohort was stratified based on a multitude of demographic and behavioral characteristics, to further elucidate the interplay between iron intake and HPV infection, we conducted an extensive series of subgroup analyses. Our cohort was stratified based on a multitude of demographic and behavioral characteristics, age, race, poverty-to-income ratio, education level, marital status, body mass index, diabetes history, alcohol consumption, history of smoking, age of first sex, and number of sexual partners (Figure 3). The results of these analyses revealed that marital status served as a notable modifier of the association between iron intake and HPV infection (*p* for interaction = 0.038). Conversely, the interaction tests performed that no significant statistical differences emerged in the connection between iron consumption and HPV infection among the other subgroups, suggesting that the differences noted among these subgroups did not have a notable impact on this inverse relationship. (*p* for interaction >0.050).

## 4 Discussion

Our cross-sectional study, the first to explore the correlation between iron intake and human papillomavirus (HPV) infection, has elucidated a pronounced L-shaped correlation, indicative of an inverse trend between the two variables. Utilizing a rigorous threshold effect analysis, we identified a pivotal inflection point at an iron intake of 16.99 milligrams. Below this threshold, we observed a statistically significant 3.2% decrease in the odds ratio (OR) of HPV infection for every incremental unit of iron consumed (OR = 0.968, 95% CI: 0.956–0.980, *p* < 0.001). Conversely, above this inflection point, the statistical significance of the correlation between iron intake and HPV infection diminished. Furthermore, our subgroup analyses provided additional evidence supporting the robustness of our findings. In conclusion, our results demonstrate a clear L-shaped correlation between iron intake and HPV infection, emphasizing that maintaining an adequate intake of iron offers protection against HPV infection.

Despite the paucity of studies investigating the association between dietary iron intake and human papillomavirus (HPV) infection, available scientific research hints at a relationship between serum iron concentrations and the underlying causes of diseases that affect the female reproductive system. Specifically, alterations in iron metabolism have been implicated in the pathogenesis of endometriosis, with ferroptosis potentially serving as a natural regulatory mechanism for the clearance of ectopic endometrial cells in healthy individuals. Perturbations in this process may contribute to the initiation and progression of endometriomas (EMs), thereby promoting their proliferation and metastatic behavior (32). Optimal maintenance of serum iron concentrations in gestational females has been established to diminish the incidence of an array of pregnancy-associated complications, encompassing anemia, hypertensive disorders of pregnancy, fetal growth restriction leading to low birth weight, preeclampsia, and various postpartum complications (33). Moreover, an investigation encompassing 2088 Iranian subjects examining dietary iron intake and cervical neoplasia suggested that adequate iron ingestion may confer a protective effect against cervical cancer incidence and potentially attenuate its progression (29). These observations are consonant with our research findings, which demonstrate an inverse relationship between dietary iron intake and HPV infection. In contrast to prior scholarly endeavors, our investigation offers nuanced elucidation of the L-shaped association between dietary iron ingestion and human papillomavirus (HPV) infection, disclosing the presence of a saturation threshold in the relationship between iron intake and HPV acquisition.

The existing body of evidence concerning the correlation between iron intake and HPV infection remains inconclusive. Prior investigations have emphasized the pivotal role of iron homeostasis in immune modulation. Initially, iron is indispensable for the differentiation and growth of epithelial tissue, which acts as the primary line of defense for the cervical mucosa against HPV infection (34). Furthermore, iron may inhibit HPV viral replication by enhancing the synthesis of interferon-gamma (IFN-*γ*), thereby amplifying IFN-γ-induced innate immune responses and mitigating the susceptibility to intracellular infection (34–36). In the realm of innate immunity, neutrophils rely on iron for the generation of reactive oxygen species (ROS) to eradicate pathogens (37). Additionally, iron plays a crucial role in promoting macrophage polarization, which is essential for cytotoxic and pro-inflammatory functions (38–40). Moreover, iron is closely associated with natural killer (NK) cells, and suboptimal iron levels impair NK cell activation and antiviral activity (41). In adaptive immunity, T cells stimulate the production of transferrin receptor (TFR) through the interleukin-2 (IL-2) signaling pathway, thereby accelerating cellular iron uptake (42–45).

We hypothesize that as iron uptake increases, T cells accumulate more iron, leading to enhanced differentiation and proliferative activity of T helper (Th) cells. This occurs by fostering cytokine production and activating the phosphorylation of cell proliferation-regulating factors via protein kinase C. Th cells further differentiate into Th1 and Th2 subsets. Th1 cells are inclined to initiate a response against intracellular viruses, and their production of IL-2 promotes lesion cell apoptosis, exerting antiviral and antitumor effects. High concentrations of these cytokines should facilitate the clearance of HPV infection and prevent lesion progression. Conversely, Th2 cells secrete various other interleukins (such as IL-4 and IL-10) and trigger immune responses against extracellular microbes (34, 36). Notably, inadequate iron intake inhibits DNA synthesis in Th1 cells, which are more sensitive to iron than Th2 cells, suggesting that insufficient iron intake has a greater inhibitory impact on Th1 than on Th2 (45). Thus, adequate iron intake supports pro-inflammatory Th1 cytokine-mediated immune responses, maintains effective immunity, and avoids a shift to anti-inflammatory Th2 cell-mediated immune responses, which could elevate the risk of extracellular infections (35, 36).

Another plausible mechanism proposes that iron possesses antiviral properties. Previous studies have demonstrated that iron can inhibit HCV viral RNA and protein expression by suppressing the enzymatic activity of RNA polymerase NS5B (47–49). However, no specific studies have examined the role of iron in HPV infection. The life cycle of HPV begins with infection of the basal cell layer following microtrauma to the epithelial barrier (50). Following the establishment of infection, the HPV genome maintains a low copy number. Upon differentiation of the host cell, HPV replicates extensively and expresses the capsid-forming genes, L1 and L2, enabling the creation and extrusion of novel viral particles from the epithelial barrier (51). To sustain a chronic infection, HPV requires the colonization of basal cells exhibiting stem-cell-like attributes (52). Therefore, cervical migratory epithelial cells become crucial targets for HPV infection, and infected cervical cells must enter the mitotic (M) phase for persistent HPV infection to occur (53). Iron-mediated ROS generation blocks the PI3K/AKT and JAK/STAT3 pathways and activates P38 MAPK, resulting in failed sustained cell division through G1 phase blockade and autophagy induction, thereby reducing the ability of HPV to sustain replication in recipient cells (54). Previous studies have also shown that ferritin can inhibit G1 cell cycle protein-dependent kinases, thus inhibiting cell division and tumor progression during growth arrest in human breast cancer cells (55). Furthermore, ingested iron is absorbed and chelated with lactoferrin or other transferrin proteins, potentially blocking viral entry into the host cell by inhibiting cellular receptors or directly binding to viral particles, which may play a role in blocking early stages of infection (56–58).

Employing the L-shaped curve model, we ascertained that 16.99 milligrams represented the inflection point in iron intake. Beyond this level, the reduction in HPV infection did not continue to increase with higher iron intake. Iron overload suppressed the proliferation rate, cell number, and active state of helper T cells (CD4+), while elevating the CD8 to CD4 ratio, promoting the production of cytotoxic T cells, and disrupting the immunoglobulin secretion pattern. This further led to an increase in the concentrations of the cytokines IL-4, IL-6, and IL-10 (59). IL-6 enhances the ability of HPV-infected cells to evade T-cell immune responses, and at high concentrations, may be crucial for the persistence of high-risk HPV (HR-HPV) infection and disease progression (45). Increased secretion of the anti-inflammatory cytokine IL-10 in the Th2 response has been associated with impaired innate and adaptive immune defenses and the progression of cervical lesions during high-risk HPV infection (60). Iron modulates macrophage activity, resulting in a reduction of TNF-*α* production, a key pro-inflammatory cytokine, which subsequently dampens the immune defense against HPV in the skin and mucosa (61).

Therefore, chronic iron supplementation and iron overload states may attenuate Th1 cytokine-mediated pro-inflammatory responses, increase Th2 cytokine-mediated anti-inflammatory responses, impair macrophage killing of intracellular pathogens, and promote the growth of pathogens due to the growth-promoting effects of iron, leading to increased susceptibility to infection. Continued excessive iron intake may disrupt the balance between Th1 and Th2, resulting in alterations in cytokine concentration and quantity, which may weaken the body’s immune response to HPV. These changes correspond to a turning point in the L-shaped curve, and the protective effect against HPV infection gradually plateaus with further increases in iron intake.

Our survey possesses several notable strengths. Foremost among these is its pioneering utilization of the NHANES database to examine the correlation between iron intake and HPV infection. The NHANES database annually enrolls a demographically diverse and representative cohort of approximately 5,000 participants from all regions of the United States, spanning various socioeconomic statuses, age strata, and ethnic groups. By harnessing nearly 12 years of extensive research data, our study encompasses a substantial sample of 7,819 women, thereby greatly enhancing the statistical power and reliability of our findings. Additionally, our investigation incorporated subgroup analyses to offer an enhanced understanding of the interplay between iron intake and HPV infection across various subgroups. Hence, our findings gain further credibility. Our investigation encounters several constraints that necessitate careful consideration. Primarily, the cross-sectional methodology employed hinders our ability to conclusively determine the temporal dynamics between iron consumption and HPV infection. Consequently, our findings can only indicate a simultaneous association between these variables, which could be affected by a wide array of additional factors. Furthermore, the cross-sectional nature of our study limits the extent to which we can capture the extended impacts of iron intake on HPV infection over time. Secondly, the exclusive focus on the U.S. population introduces potential limitations to the generalizability of our results. Significant variations in dietary patterns, HPV prevalence rates, and genetic compositions within this cohort may restrict the applicability of our findings to other demographic groups. Thus, when disseminating our results, caution should be exercised in relation to other populations. Moreover, despite adjusting for numerous covariates, limitations in the available data prevented us from comprehensively excluding other potential confounding variables, such as genetic polymorphisms and lifestyle factors, which may independently affect both iron intake and HPV infection. These unaccounted confounders may obscure or distort the true underlying relationship between the two variables. Additionally, while the NHANES data are collected through standardized personal interviews and examinations, the use of dietary recall interviews inherently involves the risk of recall bias. Factors such as participants’ memory accuracy, cognitive functioning, cultural influences, and individual dietary practices can all impact the reliability of dietary information obtained through such interviews.

To further elucidate the causal relationship between iron intake and HPV infection, future research endeavors should consider adopting longitudinal designs or cohort studies. These methodologies can provide a more precise understanding of the temporal sequence and causal links between these variables, thereby advancing our knowledge in this area.

## 5 Conclusion

Upon completion of our study, we revealed an L-shaped link between iron intake and HPV infection, with <16.99 mg/day associated with higher HPV risk. Adequate iron intake emerged as a protective factor. These findings pave the way for future research in HPV management and treatment, emphasizing iron’s crucial role in HPV pathogenesis.

## Data Availability

The original contributions presented in the study are included in the article/supplementary material, further inquiries can be directed to the corresponding author.
